# Whole-Genome Sequence of Bioactive Compound-Producing Pseudomonas aeruginosa Strain LV

**DOI:** 10.1128/MRA.01120-20

**Published:** 2021-01-07

**Authors:** Ane Stéfano Simionato, Bárbara Gionco Cano, Miguel Octavio Pérez Navarro, Eliandro Reis Tavares, Renan Augusto Ribeiro, Mariangela Hungria, Lucy Megumi Yamauchi, Sueli Fumie Yamada-Ogatta, Galdino Andrade

**Affiliations:** a Department of Microbiology, Universidade Estadual de Londrina, Londrina, Paraná, Brazil; b Soil Biotechnology Laboratory, Embrapa Soja, Londrina, Paraná, Brazil; University of Arizona

## Abstract

Pseudomonas aeruginosa is known for a high adaptive capacity due to the ability to synthesize several compounds that give advantages for competing with other microorganisms in the environment. The LV strain synthesizes bioactive compounds, mainly by secondary metabolism, with antitumor and antimicrobial activities against microbial pathogens.

## ANNOUNCEMENT

The extraordinary metabolic and physiological versatility of microbial species of the *Pseudomonas* genus allows them to live in large populations in the most diverse environments, in which they can interact with plants, animals, and humans. The plasticity of *Pseudomonas* strains makes them important agents for use in biotechnological applications (1, 2). Pseudomonas aeruginosa strains can produce a variety of polymers and secondary metabolites, which are widely used in agriculture and medicine (3–10). Here, we report the draft genome sequence of P. aeruginosa strain LV, which was isolated from an old citrus canker lesion on orange leaves (Citrus sinensis cv. Valencia), in Astorga, Brazil (23130′29.1100S, 5139′47.2000W) (11). The P. aeruginosa LV strain was grown overnight at 28°C on nutrient agar, and one colony was used for DNA extraction. The DNeasy blood and tissue kit (Qiagen, Germany) was used for genomic DNA extraction according to the manufacturer’s standard protocols. The LV strain genome was sequenced on the MiSeq platform at Embrapa Soja in Londrina, Brazil. The library was assembled using the Nextera XT DNA library preparation kit (Illumina, USA) according to the manufacturer's specifications. Paired-end reads obtained by shotgun sequencing yielded 3,378,198 sequences, with genome coverage of 150-fold. A *de novo* genome assembly was constructed with SPAdes v3.11.1 (12) after filtering and trimming reads to a quality score of >20 with CLC Genomics Workbench v20.0.4 (Qiagen). The assembly was 72 contigs, with a total length of 6,468,334 bp, an *N*_50_ value of 221,502 bp, an *L*_50_ value of 9, and a total GC content of 66.4%. The genome was analyzed on the Rapid Annotation using Subsystems Technology (RAST) v2.0 server (http://rast.nmpdr.org) (13). The RAST annotation was used for subsequent analyses, but the public genome was annotated with the NCBI Prokaryotic Genome Annotation Pipeline (PGAP) (14). The RAST annotation identified 6,015 DNA coding sequences, with 52% classified in 3,094 subsystems. The major categories were amino acids and derivatives (23.3%), carbohydrates (15.2%), and cofactors, vitamins, prosthetic groups, and pigments (11.9%). Secondary metabolite biosynthesis genes were identified by using antiSMASH v4.1 (https://antismash.secondarymetabolites.org) (15), and 16 putative gene clusters responsible for secondary metabolite biosynthesis were identified. Among them, we can highlight genes related to expression of the β-lactone (thanamycin), bacteriocin, thiopeptide, nonribosomal peptide synthetase cluster (pyochelin, pyoverdin, and rhizomide), and phenazine (pyocyanine and streptophenazine) types. The acquired antibiotic resistance genes were identified using ResFinder v3.2 (https://cge.cbs.dtu.dk/services/ResFinder) (16); they were related to resistance to quinolones (*crpP*), β-lactams (*bla*_OXA-396_, *bla*_OXA-486_, and *bla*_PAO_), aminoglycosides [*aph(3′)-llb*], phenicols (*catB7*), and fosfomycin (*fosA*). With CRISPRfinder (https://crispr.i2bc.paris-saclay.fr) (17), we found three clustered regularly interspaced short palindromic repeat (CRISPR) arrays. The genome of P. aeruginosa strain LV may facilitate understanding and exploration of metabolic pathways in the search for potential bioactive compounds.

### Data availability.

This whole-genome shotgun project has been deposited in DDBJ/ENA/GenBank under the accession no. CP058323 (BioProject no. PRJNA450135, BioSample no. SAMN08930812, and SRA accession no. SRR13065837). The version described in this paper is the first version.
